# Negative body image: Relationships with heightened disgust propensity, disgust sensitivity, and self-directed disgust

**DOI:** 10.1371/journal.pone.0198532

**Published:** 2018-06-05

**Authors:** Paula von Spreckelsen, Klaske A. Glashouwer, Elise C. Bennik, Ineke Wessel, Peter J. de Jong

**Affiliations:** 1 Department of Clinical Psychology and Experimental Psychopathology, University of Groningen, Groningen, The Netherlands; 2 Center for Eating Disorders, Accare, Child and Adolescent Psychiatry, Groningen, The Netherlands; Wayne State University, UNITED STATES

## Abstract

Consistent with the view that disgust might be involved in persistent body dissatisfaction, there is preliminary evidence showing a positive correlation between measures of negative body image and indices of both trait disgust and self-directed disgust. In two correlational studies among undergraduates (*N* = 577 and *N* = 346, respectively) we aimed at replicating and extending these findings by testing a series of critical relationships, which follow from our hypotheses that 1) trait disgust propensity would increase the risk of developing a negative body image by increasing the likelihood of feeling self-disgust, and 2) trait disgust sensitivity would heighten the impact of self-disgust on the development of persistent negative body appraisals. Replicating previous research, both studies showed that negative body image was positively related to self-disgust, disgust propensity and disgust sensitivity. Mediation analyses showed that, in line with our model, self-disgust partly accounted for the association between disgust propensity and negative body image. Although disgust sensitivity showed an independent relationship with body image, disgust sensitivity did not moderate the association between self-disgust and negative body image. All in all, findings are consistent with the view that self-disgust-induced avoidance may contribute to persistent negative body appraisals.

## Introduction

Within modern western society, many people, in particular girls and women, have a negative body image [1]. *Body image* is a complex construct encompassing thoughts, behaviors, feelings and evaluations related to one’s body [2]. A negative body image may manifest itself by a strong importance of, as well as preoccupation and dissatisfaction with one’s shape and weight. Research has mainly focused on this latter affective aspect of body image (body dissatisfaction) and provided support for its persistence [3] and its association with general public health concerns (e.g., decreased quality of life related to physical health, lower mental health, and obesity; [4–6]). In addition, it has been found that body dissatisfaction is a critical factor in the development, maintenance, and relapse of eating disorders (e.g., [7–9]). In order to tackle the negative implications associated with a negative body image, it is critical to improve our insight in the mechanisms that may be involved in its development and persistence.

The exposure to societal pressures related to body shape and weight (e.g., media promoting thin bodies; objectification of female bodies) and to aversive experiences (e.g., childhood trauma; body-related bullying) have been identified as risk factors for the development of a negative body image/body dissatisfaction [9, 10]. Such experiences have been proposed to promote an internalization of a critical, highly negative, distorted, and even repulsive way of relating to one’s own body [11]. In line with the latter idea, the risk factors associated with a negative body image have also been implicated in the formation of body-directed *self-disgust*, a self-evaluation marked by the general appraisal of one’s own body parts as revolting ([12]; self-disgust is referring to the appraisal of parts of the self, which can be physical or characterological/behavioral; in the present study, we focus on physical/body-related self-disgust). While preliminary evidence suggests that self-disgust and body image disturbances are associated [13], an important next step would be to elucidate which factors play a role in the relationship between self-disgust and negative body image.

The experience of disgust in response to repulsive stimuli (e.g., pathogens, unfit sexual partners, moral transgression) is characterized by a distinctive facial expression (wrinkled nose, etc.), a specific subjective experience (nausea), and behavioral/cognitive avoidance. Previous research has found that people with a relatively strong habitual inclination to respond with disgust to any given stimulus or situation (i.e., high *disgust propensity*) are more likely to exhibit self-disgust [14, 15]. In response to the risk factors mentioned above (i.e., social pressures and aversive experiences), high disgust propensity may make people more likely to experience disgust towards own bodily aspects (e.g., body fat, acne). In response to repeatedly experiencing self-directed disgust, a stable schematic construct of the own body as repulsive may develop.

In line with the behavioral manifestation associated with any form of disgust, the self-disgust schema is assumed to serve the ultimate goal of avoiding exposure to the disgust-eliciting stimulus, which is in this case the own body. Avoidance has been shown to perpetuate associations of disgust, making these associations particularly resistant to extinction [16]. Avoidance of body-related situations (e.g., mirror exposure) or mental experiences (e.g., thinking about one’s body) might prevent: (1) appreciation of attractive aspects of one’s body, and (2) habituation to and re-evaluation of ‘aversive’ (disgust eliciting) body parts. As a result, access to critical information that could counteract pre-existing negative associations or help to develop positive body-related associations might become blocked. Over time, avoidance behaviors might contribute to the aggravation of negative body-related emotions, behaviors and cognitions (i.e., negative body image). In sum, high disgust propensity may render people susceptible to develop a stable appraisal of the own body as disgusting, which, in turn, might contribute to the development/reinforcement of a negative body image.

Previous research emphasized the importance of making a distinction between trait disgust propensity (tendency to experience disgust more readily) and trait *disgust sensitivity*, which is the tendency to find the emotion of disgust unpleasant [17, 18]. Recent research showed a positive association between disgust sensitivity and self-disgust [13]. Theoretically, the relationship between self-disgust and negative body image would be expected to be especially pronounced in individuals who show a relatively strong disgust sensitivity. Higher levels of disgust sensitivity are thought to motivate people to avoid stimuli that are expected to evoke the unpleasant emotion of disgust [17]. Thus, self-disgust induced avoidance of one’s body would be relatively high in individuals with a strong general habitual tendency to avoid exposure to disgust elicitors (high disgust sensitivity), which in turn would contribute to the amplification of a negative body image (cf. [19]).

**Fig 1:**
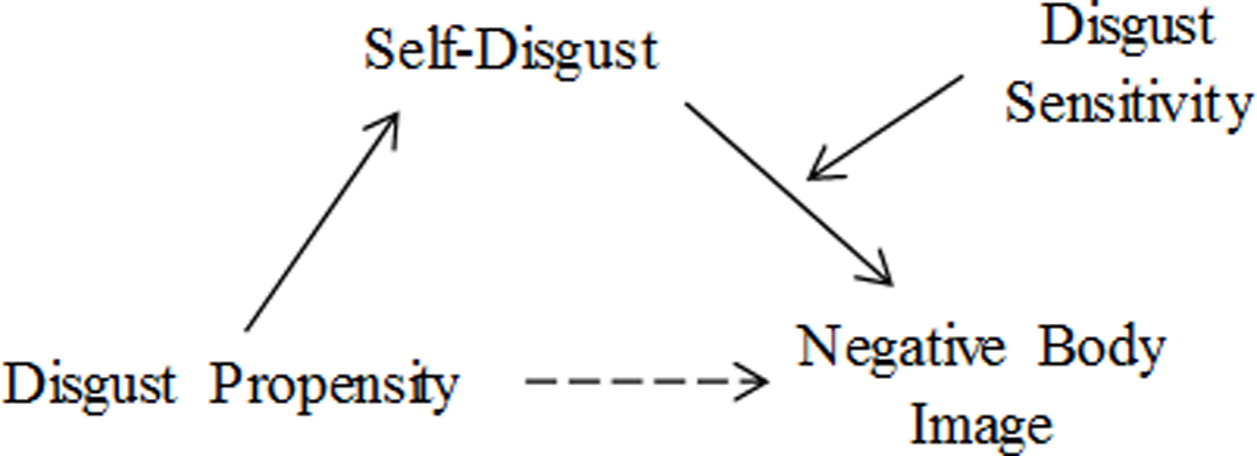
Hypothesized model of how disgust propensity and disgust sensitivity may differentially promote the relationship between self-disgust and a negative body image.

On the basis of the available evidence, we propose a model that helps explain how the different forms of disgust may contribute to the development and persistence of a negative body image (Fig 1). Specifically we propose that: (1) disgust propensity increases the likelihood for people to have a negative body image by making them more liable to experiencing self-disgust, and (2) disgust sensitivity moderates the association between self-disgust and negative body image. Although beyond the scope of this investigation, we acknowledge the importance of external factors, like societal pressures or aversive personal experiences, which may affect the interrelationships presented in our model (e.g., influencing the association between disgust propensity and self-disgust).

Unfortunately, the research on the role of disgust in the experience of a negative body image is quite sparse. However, there is some indirect support for the relationships between self-disgust, trait disgust and negative body image from the literature on eating disorders. In short, research found that patients with eating disorders not only tend to show body image disturbances [20], but also self-disgust [21, 22] and self-disgust related avoidance [23]. In addition, it has been found that disgust propensity and disgust sensitivity are increased in patients with an eating disorder, and positively associated with eating disorder symptoms [24–27]. In sum, those findings contribute to the empirical foundation for our model.

## Study 1

**Fig 2:**
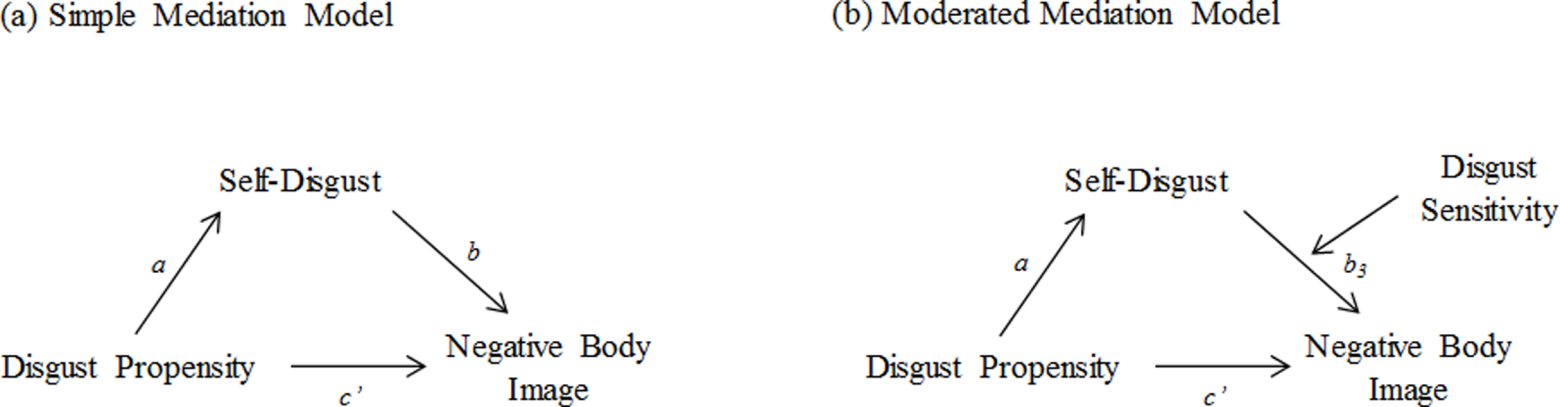
Illustration of the statistical analyses.

In study 1, we first tested the robustness of previous findings showing relationships between trait disgust propensity and sensitivity, self-disgust and negative body image. Subsequently, we investigated whether self-disgust would mediate the association between disgust propensity and negative body image by conducting a simple mediation analysis (Fig 2A). As a second step, we tested whether the association between self-disgust and negative body image was stronger for people with high disgust sensitivity by extending the simple mediation analysis to a moderated mediation analysis (Fig 2B).

## Method

### Participants and procedure

The sample of the first study consisted of 577 first year female psychology students of the University of Groningen who took part in an online survey. Participants ranged in age from 17 to 29 years (*M* = 19.63; *SD* = 1.65) and had a mean self-reported BMI of 21.58 (*SD* = 3.06). The survey was administered during the first semester of two subsequent student cohorts (fall 2013/fall 2014). Students received course credits in exchange for participation. The study (incl. the inclusion of participants aged 17 without parent/guardian consent) was approved by the Ethics Committee Psychology of the University of Groningen. Because the study was part of a large scale screening aiming at optimizing power, the power levels were high (1.0) to detect medium effect sizes (*f*^*2*^ = .15) in both the simple and moderated mediation analyses (to detect *R*^*2*^ deviation from zero in multiple regression with 2 and 4 predictors, respectively). The power levels for detecting small effect sizes (*f*^*2*^ = .02) were 0.87 (simple mediation) and 0.78 (moderated mediation). The power calculations were conducted in G*power 3.1.9.

### Materials

#### Shape and weight concern subscales of the eating disorder Examination-questionnaire (EDE-Q)

Negative body image was measured by combining the weight and shape concern subscales of the most recent version of the EDE-Q (EDE-Q 6.0; [28]). The subscales include items assessing the affective-evaluative (e.g., body dissatisfaction, fear of gaining weight) and cognitive-behavioral (e.g., importance of and preoccupation with shape/weight) dimensions of body image defined by Cash [2]. Items are answered on a scale between 0 (no days) and 6 (every day). The combined weight and shape concern subscales showed high internal consistency within this study (*α* = .94).

#### Disgust propensity and sensitivity scale—revised (DPSS-R)

The DPSS-R [29] consists of 8 items measuring disgust propensity (i.e., the tendency to experience disgust in a wide variety of situations) and 8 items measuring disgust sensitivity (i.e., how awful do participants consider this disgust experience). Items are rated on a 5-point scale from 1 (= ‘never’) to 5 (= ‘always’) with total scores ranging from 16 to 80. The DPSS-R and its subscales have shown to be internally consistent with alphas > .71 (e.g., [17, 18]). In the present study, internal consistency was good for both propensity (*α* = 0.80) and sensitivity (α = 0.77).

#### Self-disgust scale (SDS)

Self-disgust was measured with the SDS [15], which consists of 18 items (e.g., “I find myself repulsive”) that are rated on a 7-point Likert scale (1 = strongly agree, 7 = strongly disagree). The scale contains six neutral filler statements. A total self-disgust score was found by summing scores to the remaining 12 statements, after reverse coding items 1, 3, 4, 7, 10, 12, 15, 17 and 18. Total scores range from 12 to 84. In the present study, internal consistency was high (*α* = 0.91).

## Results

### Descriptives

Table 1 presents the means, standard deviations and inter-correlations for all variables in the analyses (disgust propensity & sensitivity, self-disgust, body image). We noticed no strong normality violations in the distribution of the variables.

**Table 1:** Means, standard deviations and inter-correlations for all variables of the analysis, *N* = 577.

	*M*	*SD*	1.	2.	3.
1. Disgust Propensity^a^	20.74	4.62	-		
2. Self-disgust^b^	33.12	12.23	.13*	-	
3. Disgust Sensitivity^c^	17.02	4.88	.62*	.24*	-
4. Negative Body Image^d^	1.95	1.45	.15*	.40*	.21*

^a^Disgust propensity: disgust propensity subscale of the DPSS-R;

^b^Self-disgust: SDS;

^c^Disgust sensitivity: disgust sensitivity subscale of the DPSS-R;

^d^Negative body image: composite score of shape and weight concern subscales of the EDE-Q;

* = *p* < .05.;

#### Bivariate correlations

All bivariate correlations were statistically significant. The strongest association was found between disgust propensity and disgust sensitivity (*r* = .62), as expected given previous research [30]. Similarly, the association between self-disgust and negative body image was quite strong (*r* = .40), thus validating previous research [13]. Also the correlation between self-disgust and disgust propensity was significant although this relationship was relatively weak (*r* = .13). Overall, we found support for significant bivariate associations between disgust propensity, disgust sensitivity, self-disgust, and negative body image.

### Mediation analyses

Inspection of regression residuals for both mediation analyses revealed no violations of assumptions (linearity, homoscedasticity, normality; see S1 Appendix). All mediation analyses were conducted in PROCESS version 3 [31].

#### Simple mediation

A simple mediation analysis was conducted to test whether the relationship between disgust propensity (predictor) and negative body image (outcome variable) can be accounted for by self-disgust (mediator; see Fig 2A for a depiction of the simple mediation model). The results of the mediation analysis are displayed in Table 2. Regressing disgust propensity on negative body image revealed a significant total effect (path *c*; *b* = .046), which remained significant, but decreased in strength in the full model (direct effect; path *c’*; *b* = .029). In addition, disgust propensity significantly predicted self-disgust (path *a*; *b* = .351), which was a significant predictor of negative body image (path *b*; *b* = .046). The indirect effect of disgust propensity on negative body image (path *ab*; effect: .016) yielded a bias-corrected bootstrap confidence interval that did not include zero (.005 –.029), indicating that self-disgust mediated the relationship between disgust propensity and negative body image. Calculating the percent mediation (*P(m*) = ab/c = .016/.046) showed that 34.78% percent of the total effect was accounted for by the indirect effect, thus implying that self-disgust was able to account for part of the relationship between disgust propensity and negative body image.

**Table 2:** Simple mediation analysis with disgust propensity and self-disgust (mediator) on negative body image.

	Path/effect	*B*	*SE*	*t*	*p*	*CI*
**Simple Regression Models**						
*R*^*2*^ = .018; *F* (1, 575) = 12.33; *p* < .001	*c* (total effect of DP^1^ on NBI^2^)	.046	.013	3.51	< .001	.020 –.071
*R*^*2*^ = .021; *F* (1, 575) = 10.28; *p* = .001	*a* (DP on SD^3^)	.351	.109	3.21	< .001	.136 –.566
**Multiple Regression Model**						
*R*^*2*^ = .170; *F* (2, 574) = 58.71; *p* < .001	*c’* (direct effect of DP on NBI)	.029	.012	2.43	.015	.006 –.053
	*b* (SD on NBI)	.046	.005	10.14	< .001	.037 –.055
		**Effect**	***Boot***^***4***^ ***SE***			***Boot***^***4***^ ***CI***
	*ab* (indirect effect of DP on NBI through SD)	.016	.006			.005 –.029

^1^ DP = Disgust propensity

^2^ NBI = Negative body image

^3^ SD = Self-disgust

^4^ Boot = bias-corrected bootstrap standard error/confidence interval

#### Moderated mediation

To test whether disgust sensitivity would moderate the effect of self-disgust on negative body image, we extended the simple mediation model to a moderated mediation analysis (see Fig 2B for a depiction of the moderated mediation analysis). As can be seen in Table 3, the interaction between self-disgust and disgust sensitivity did not significantly predict negative body image (path *b*_*3*_; *b* = -.001), implying that disgust sensitivity did not moderate the association between self-disgust and negative body image. This is in line with the finding that the bias-corrected bootstrap confidence interval of the index of moderated mediation included zero (-.001 –.000; see last row in Table 3). Thus, we did not find support that disgust sensitivity moderates the relationship between self-disgust and negative body image. In contrast to the simple mediation model tested above, disgust propensity was no longer significantly associated with negative body image in the current model. This finding is, however, more likely due to the inclusion of disgust sensitivity in the model (which was a significant predictor of negative body image [path *b*_*2*_; *b* = .030] and was strongly associated with disgust propensity [see Table 1]), than to self-disgust attaining a full mediation effect.

**Table 3:** Moderated mediation analysis with disgust propensity, self-disgust (mediator), disgust sensitivity (moderator) on negative body image.

	Path/effect	*B*	*SE*	*t*	*p*	*CI*
See Table 2 for pathways/effects *c* (total effect of DP^1^ on NBI^2^) and *a* (DP on SD^3^)						
**Multiple Regression Model**						
*R*^*2*^ = .176; *F* (4, 572) = 30.60; *p* < .001	*c’* (direct effect of DP^1^ on NBI^2^)	.011	.015	.718	.473	-.020 –.041
	*b*_*1*_ (SD^3^ on NBI)	.044	.005	9.56	< .001	.035 –.053
	*b*_*2*_ (DS^4^ on NBI)	.030	.015	2.02	.044	.001 –.060
	*b*_*3*_ (SD x DS on NBI)	-.001	.001	-.841	.401	-.002 –.001
		**Effect**	***Boot***^***5***^ ***SE***			***Boot***^***5***^ ***CI***
	Index of moderated mediation	-.000	.000			-.001 –.000

^1^ DP = Disgust propensity

^2^ NBI = Negative body image

^3^ SD = Self-disgust

^4^ DS = Disgust Sensitivity

^5^Boot = bias-corrected bootstrap standard error/confidence interval

## Study 2

It should be acknowledged that the measure of self-disgust that was used in study 1 might have been suboptimal for capturing some critical aspects of self-disgust in the context of body image disturbances. In line with this, Moncrieff-Boyd, Allen, Byrne, and Nunn [13] recently developed a modified version of the SDS, The *Self-Disgust Eating Disorder Scale* (SDES) to assess self-disgust in the context of eating pathology. Specifically, the modified scale aims to assess the visceral quality of self-disgust conceptualized as revulsion towards ones’ own body and self and low acceptance of the self in order to improve its distinction from other related concepts (e.g., self-esteem). Utilizing the less optimal measure of self-disgust might have contributed to the lack of full mediation found in Study 1.

In addition, the elicitor-independent measure of disgust propensity might also have been suboptimal for testing our model. The DPSS-R was designed as a domain-independent index of disgust propensity. It is, however, possible that it is not the overall liability to experience disgust that is most critical in the context of negative body image, but the tendency to experience disgust for particular classes of stimuli. Recent conceptualizations of disgust distinguish between three core forms of disgust [32]. *Pathogen disgust* refers to disgust in response to primary disgust elicitors that are associated with contamination or illness (e.g., rotten food, body products). *Sexual disgust* refers to disgust in response to the prospect of having sex with suboptimal sex partners. *Moral disgust* can be elicited by acts that represent moral transgressions of socio-cultural norms [33].

Especially with regard to pathogen and sexual disgust there seem to be obvious pathways explaining how these propensities might relate to a negative body image. Aspects of the body, for example acne or body-fat, may elicit disgust by both representing signs of illness (pathogen disgust) and suboptimal mating quality in terms of genetic makeup (sexual disgust). Those people with a strong tendency to experience pathogen or sexual disgust in response to bodily features in general, may be more likely to also experience disgust in response to own bodily features = self-disgust) and thus more likely to hold a negative body image. Lastly, moral disgust may play a role in the context of a negative body image when parts of the own body are seen as violations of common standards of beauty. However, since cultural standards of beauty are not a central aspect of moral disgust, and because moral disgust is more related to evaluations emanating from others rather than the self, we anticipated that its association with self-disgust/a negative body image would be relatively weak

Study 2 was designed to examine the relevance of each of the disgust propensity domains in the experience of a negative body image. The distinction between different forms of disgust might help us to shed light on the involvement of self-disgust in the association between trait disgust and negative body image. To further enhance the sensitivity of the current study to test whether indeed self-disgust mediates the relationship between disgust propensity and negative body image, we used the modified self-disgust scale (SDES). Thus, study 2 was designed to investigate the relationships between different classes of disgust propensity and both self-disgust (as indexed by the SDES), and negative body image, as well as the relationship between self-disgust and negative body image.

As a first step, we aimed to investigate the stability of the results obtained in study 1 by using a different sample and an alternative measure of self-disgust. Thus, we examined whether eating disorder related self-disgust (SDES) would mediate the association between disgust propensity and negative body image (simple mediation analysis), and whether disgust sensitivity would moderate the association between self-disgust (SDES) and negative body image (moderated mediation analysis). As a novel next step, we conducted the same analyses (simple mediation & moderated mediation), but substituted general disgust propensity with the different classes of disgust propensity as (separate) predictors in the analyses.

## Method

### Participants and procedure

The sample of the second study consisted of 346 students (69 men, 277 women) of the University of Groningen. Participants were recruited in several ways: i) 151 participants were first year psychology students who received course credits in exchange for participation; ii) 195 participants were recruited via Facebook or a paid participant pool. The second group could win a voucher of €20 in exchange for participation (20 vouchers were raffled). Participants ranged in age from 17 to 31 years (*M* = 21.14; *SD* = 2.51) and had a mean BMI of 22.07 (*SD* = 3.65). The survey was administered during the first semester of one student cohort (fall 2015). The rest of the design was identical to Study 1. The study was approved by the Ethics Committee Psychology of the University of Groningen. Similar to study 1, the power levels in study 2 were high (1.0) to detect medium effect sizes (*f*^*2*^ = .15) in both the simple and moderated mediation analyses (to detect *R*^*2*^ deviation from zero in multiple regression with 2 and 4 predictors, respectively). The power levels for detecting small effect sizes (*f*^*2*^ = .02) were 0.65 (simple mediation) and 0.53 (moderated mediation).

### Materials

The same questionnaires as in Study 1 were administered. Again, the scales showed high internal consistency (combined EDE-Q weight and shape concern subscales: *α* = .94; DPSS propensity: *α* = 0.81; DPSS sensitivity: *α* = 0.78; SDS: *α* = 0.93).

#### Self-disgust eating disorder scale (SDES)

In addition to the original SDS [15], the SDES [13] was administered. The wording of some original items was altered with the goal of capturing a more precise definition of self-disgust as visceral revulsion at the self. In addition, 4 items were excluded and two items were added to the scale in order to assess disgust of the body. In the process of translating the original English version into Dutch, we decided to adjust one of the items (in collaboration with Dr. Moncrieff-Boyd; Original: “When I walk around, I feel revolting”; Adjusted: “I think that other people are disgusted by me”) because a direct translation into Dutch would have been ambiguous and odd. The revised version of the scale comprises 16 items to be rated on a 7-point Likert scale (1 = strongly agree, 7 = strongly disagree). Six filler items are removed for scoring giving 10 items for use in score calculations (items 1, 3, 6, 9, 11, 13 and 16 are reverse-scored). Scores can range from 10 to 70, with higher scores indicating greater levels of self-disgust. In the present study internal consistency was high (*α* = 0.94). Furthermore, there was a high correlation between the SDS and the SDES (Spearman’s rho = .92).

#### Three domains of disgust scale (TDDS)

The TDDS [32] is a 21-item self-report measure of disgust propensity in three domains: moral disgust (e.g., stealing from a neighbor), sexual disgust (e.g., hearing two strangers having sex) and pathogen disgust (e.g., seeing a cockroach run across the floor). Items are scored on a 7-point Likert-scale ranging from not at all disgusting (0) to extremely disgusting (6). Scores of each subscale can range from 0 to 42. In the current sample the subscales showed high internal consistency (moral disgust: *α* = 0.90; sexual disgust: *α* = 0.84; pathogen disgust: *α* = 0.79).

## Results

### Descriptives

Table 4 presents the means, standard deviations and inter-correlations for all variables in the analysis (disgust propensity, the three domains of disgust propensity, disgust sensitivity, self-disgust, negative body image). The distributions of self-disgust (SDES), moral and pathogen disgust propensity were skewed in the current sample.

**Table 4:** Means, standard deviations and non-parametric inter-correlations (spearman’s rho) for all variables of the analysis, *N* = 346.

	*M*	*SD*	1.	2.	3.	4.	5.	6.
1. Disgust propensity^a^	19.62	4.95	-					
2. Moral disgust^b^	23.82	9.85	.12*	-				
3. Sexual disgust^b^	19.77	9.19	.33*	.35*	-			
4. Pathogen disgust^b^	25.01	7.39	.44*	.41*	.56*	-		
5. Self-disgust^c^	28.74	14.1	.26*	-.01	.01	.01	-	
6. Disgust Sensitivity^d^	16.42	5.00	.64*	.17*	.27*	.36*	.29*	-
7. Negative Body Image^e^	1.99	1.47	.27*	.07	.25*	.28*	.47*	.31*

^a^Disgust propensity: disgust propensity subscale of the DPSS-R.

^b^Disgust, sexual & pathogen disgust: subscales of the TDDS

^c^Self-disgust: SDES.

^d^Disgust sensitivity: disgust sensitivity subscale of the DPSS-R.

^e^Negative body image: composite score of shape and weight concern subscales of the EDE-Q.

* = *p* < .05.

#### Bivariate correlations

Due to the presence of several skewed variables, non-parametric bivariate correlations were obtained. In line with the results of study 1, we found significant positive associations between self-disgust and negative body image, disgust propensity and disgust sensitivity, as well as between negative body image and disgust propensity and sensitivity. In line with our new predictions, disgust propensity was positively associated with the three domains of disgust propensity, with the highest association with pathogen disgust and the lowest with moral disgust. Negative body image exhibited significant positive associations with sexual and pathogen disgust. This lends support to our theorized association between both sexual and pathogen disgust and negative body image. Negative body image was not significantly associated with moral disgust, which is in line with our theorized lack of an evident role of moral disgust in negative body image. In contrast to our predictions, self-disgust did not show significant associations with either domain of disgust propensity. This is surprising since self-disgust as well as the sexual and pathogen domains of disgust propensity showed separate positive associations with both general disgust propensity and negative body image.

### Conceptual replication of study 1

As in study 1, inspection of regression residuals for both mediation analyses revealed no violations of assumptions (linearity, homoscedasticity, normality; see S1 Appendix).

#### Simple mediation

The results of the simple mediation analysis with self-disgust (SDES) acting as a mediator on the association between disgust propensity and negative body image (see Table 5) were similar to those in study 1. Again, the regression coefficient of disgust propensity (on negative body image) remained significant, but decreased in strength in the full model (direct effect; path *c’*; *b* = .068) compared to the total effect model (path *c*; *b* = .085). In addition, both paths *a* (disgust propensity on negative body image; *b* = .574) and *b* (self-disgust on negative body image; *b* = .029) were significant and their product (path *ab*; indirect effect; effect: .017) yielded a bias-corrected bootstrap confidence interval that did not include zero (.007 –.030). The percent mediation (*P(m)* = ab/c = 0.017/0.085) yielded a lower estimate (20%) compared to study 1 (35%). Despite small differences, the results of study 2 are in line with study 1 by replicating the finding that self-disgust partially accounted for the relationship between disgust propensity and negative body image.

**Table 5:** Simple mediation with disgust propensity and self-disgust (SDES; mediator) on negative body image.

	Path/effect	*B*	*SE*	*t*	*p*	*CI*
**Simple Regression Models**						
*R*^*2*^ = .081; *F* (1, 344) = 30.17; *p* < .001	*c* (total effect of DP^1^ on NBI^2^)	.085	.015	5.49	< .001	.054 –.115
*R*^*2*^ = .041; *F* (1, 344) = 14.62; *p* < .001	*a* (DP on SD^3^)	.574	.150	3.82	< .001	.279 –.869
**Multiple Regression Model**						
*R*^*2*^ = .155; *F* (2, 343) = 31.37; *p* < .001	*c’* (direct effect of DP on NBI)	.068	.015	4.50	< .001	.038 –.098
	*b* (SD on NBI)	.029	.005	5.48	< .001	.017 –.040
		**Effect**	***Boot***^***4***^ ***SE***			***Boot***^***4***^ ***CI***
	*ab* (indirect effect of DP on NBI through SD)	.017	.006			.007 –.030

^1^ DP = Disgust propensity

^2^ NBI = Negative body image

^3^ SD = Self-disgust (SDES)

^4^ Boot = Bias-corrected bootstrap standard error/confidence interval

#### Moderated mediation

As in study 1, no support for the hypothesis that disgust sensitivity moderated the association between self-disgust (SDES) and negative body image was found, as evidenced by the non-significant interaction between self-disgust and disgust sensitivity (path *b*_*3*_; *b* = .001) and the bias-corrected bootstrap confidence interval of the index of moderated mediation that included zero (-.001 –.002; see Table 6) . Again, the lack of a significant association between disgust propensity and negative body image is likely due to the inclusion of disgust sensitivity in the model.

**Table 6:** Moderated mediation analysis with disgust propensity, self-disgust (SDES; mediator), disgust sensitivity (moderator) on negative body image.

	Path/effect	*B*	*SE*	*t*	*p*	*CI*
See Table 2 for pathways/effects *c* (total effect of DP on NBI) and *a* (DP on SD)						
**Multiple Regression Model**						
*R*^*2*^ = .176; *F* (4, 341) = 18.62; *p* < .001	*c’* (direct effect of DP^1^ on NBI^2^)	.030	.020	1.55	.122	-.008 –.069
	*b*_*1*_ (SD^3^ on NBI)	.028	.005	5.21	< .001	.017 –.037
	*b*_*2*_ (DS^4^ on NBI)	.056	.020	2.87	.044	.018 –.094
	*b*_*3*_ (SD x DS on NBI)	.001	.001	.488	.626	-.002 –.003
		**Effect**	***Boot SE***			***Boot***^***5***^ ***CI***
	Index of moderated mediation	.000	.001			-.001 –.002

^1^ DP = Disgust propensity

^2^ NBI = Negative body image

^3^ SD = Self-disgust (SDES)

^4^ DS = Disgust Sensitivity

^5^Boot = Bias-corrected bootstrap standard error/confidence interval

### Domains of disgust propensity

Due to the lack of significant associations of self-disgust with sexual, moral and pathogen disgust, no further mediation or moderated mediation analyses were conducted.

### Post-hoc analysis

Based on the results obtained, a hierarchical regression analysis was conducted in order to assess whether different forms of trait disgust are independently related to negative body image. The first step was to regress the three domains of disgust on negative body image. Pathogen, but neither moral nor sexual disgust, was significantly related to negative body image (see Model 1 in Table 7), which indicates that pathogen disgust exhibited a relationship with negative body image independent of the other domains of disgust propensity. Since pathogen disgust was the only significant predictor, the other two domains were excluded from the following steps of the regression analysis. As a next step, general disgust propensity was added as a predictor. Pathogen and general disgust propensity were significant and independent predictors of negative body image (see Model 2 in Table 7). As a last step, disgust sensitivity was added as a predictor. Both disgust sensitivity and pathogen disgust exhibited significant associations with negative body image independent from each other and from general disgust propensity (see Model 3 in Table 7). In sum, the results suggest that pathogen disgust was a significant predictor of negative body image independent from self-disgust (see bivariate correlation in Table 4), other domains of disgust propensity, general disgust propensity and disgust sensitivity.

**Table 7:** Stepwise regression analysis with the three domains of disgust propensity, general disgust propensity and disgust sensitivity on negative body image.

	Predictor	*β*^*a*^	*t*	*p*	*CI*
**Model 1**					
*R*^*2*^ = .081; *F* (1, 344) = 30.17; *p* < .001	Pathogen disgust	.236	3.57	<.001	.021 –.073
	Sexual disgust	.104	1.56	.113	-.004 –.037
	Moral disgust	-.065	-1.13	.261	-.027 –.007
**Model 2**					
*R*^*2*^ = .107, *F* (2, 345) = 20.6, *p*	Pathogen disgust	.181	3.20	.002	.014 –.058
	Disgust propensity	.205	3.62	<.001	.028 –.094
**Model 3**					
*R*^*2*^ = .129, *F* (3, 345) = 16.9, *p*	Pathogen disgust	.158	2.80	.005	.009 –.054
	Disgust propensity	.083	1.18	.237	-.016 –.065
	Disgust sensitivity	.200	2.95	.003	.020 –.098

^*a*^ *β* = standardized regression coefficient.

## General discussion

The main findings can be summarized as follows: Both studies consistently showed that negative body image was associated with (i) higher self-disgust, and (ii) heightened disgust propensity and disgust sensitivity. In addition, both studies showed that (iii) the relationship between stimulus-independent disgust propensity and negative body image was partly mediated by self-disgust, whereas the relationship between self-disgust and negative body image was not moderated by disgust sensitivity.

In line with our prediction and consistent with previous research, both studies showed that people who tended to experience disgust more readily (high disgust propensity) were more likely to experience disgust towards themselves (self-disgust; [14, 15]) and to report a more negative body image. Post hoc analysis indicated, however, that only disgust sensitivity showed an independent relationship with negative body image whereas the relationship of disgust propensity (as indexed by the DPSS-R) with negative body image was no longer significant when statistically correcting for disgust sensitivity.

Consistent with our heuristic model, the relationship between disgust propensity and negative body image was partly mediated by self-disgust. In other words, the repeated finding that negative body image is associated with high trait disgust can be partly explained by the relationship between disgust propensity and self-disgust. High disgust proneness may set people at risk for developing a negative body image via a heightened liability to develop self-directed disgust. However, the direct relationship between disgust propensity and self-disgust was rather small (in particular in the first study). This finding might be due to general disgust being an adaptive emotion (e.g., promoting health and survival), but self-disgust being a maladaptive construct (e.g., associated with detrimental effects on mental wellbeing; [12]). In addition, this observation could point towards the role of additional factors, next to a high propensity to experience disgust, that need to be considered in order to explain the occurrence of self-disgust. As described before, those ‘vulnerability factors’ may refer to societal influences and/or personal experiences implicated in the formation of self-disgust [12]. It might be interesting for future research to investigate if those vulnerability factors indeed have an influence on the relationship between disgust propensity and self-disgust.

The direct relationship between self-disgust and negative body image was similar for both the original (SDS) and adapted version (SDES) of the Self Disgust Scale; also the properties of being a mediator in the relationship between disgust propensity and negative body image was similar for both versions. However, the mediation effect was slightly less pronounced for the adapted version (lower percent mediation), possibly indicating that additional factors (e.g., societal influence; personal experiences) may play a larger role in the more specific context of body-related self-disgust (i.e., as assessed with the SDES). Nonetheless, the findings support both the robustness of the construct of self-disgust as well as the relationship between self-disgust and negative body image. Since the current findings are correlational in nature it remains to be tested in experimental research whether the relationship has causal properties and whether, for example, lowering self-disgust may improve one’s body image (e.g., via reducing self-disgust induced avoidance).

Although a high general disgust propensity was associated with self-disgust as well as pathogen, sexual, and to a lesser extent moral disgust propensity, no significant associations were found between self-disgust and either one of these domain specific inclinations to experience disgust. This might indicate that the propensity to experience disgust in general is involved in the formation of disgust associations with several kinds of stimuli, including pathogen, sexual and moral stimuli, as well as the self. However, disgust associations towards stimuli that extend from primary or disease-eliciting stimuli to more complex stimuli (e.g., moral acts, the self) may be relatively unrelated to those disgust associations related to primary contaminants (pathogen stimuli; cf. [34]) and potentially develop at later ages and under the influence of relevant (e.g., interpersonal) experiences.

Based on our post-hoc analysis, a tendency to experience disgust towards disease-eliciting stimuli, like spoiled food or bodily products, seems to be related to negative body image, independent of other disgust domains and general disgust propensity. In line with this observation, research indicates that eating disorders are characterized by a high tendency to feel disgust towards bodily products [25, 27, 35, 36]. It may be that a relatively strong inclination to experience disgust in response to bodily products and particular body features (high pathogen disgust) contributes to a perception of human bodies as repulsive in general, thereby making people more likely to have a negative body image. However, increased pathogen disgust may not necessarily encompass an appraisal of the own body as especially disgusting compared to other bodies (i.e., self-disgust). Nevertheless, in order to draw firm conclusions, future studies are needed to further explore the role of pathogen disgust in body image.

People who experienced feelings of disgust as particularly negative (high disgust sensitivity) were more likely to experience self-disgust as well as negative body image. This is in line with previous research demonstrating positive associations between disgust sensitivity and self-disgust [13], and between disgust sensitivity and eating disorder symptoms [24]. In contrast to our predictions, a negative appreciation of experiencing disgust was not associated with a higher likelihood to experience a negative body image in people with high self-disgust. One explanation could be that self-disgust is in itself already so aversive leaving not much room for heightened disgust sensitivity to further moderate the strength of self-disgust-induced avoidance (which in turn would lead to the persistence of a negative body image).

Although our current investigation aimed at discovering self-disgust related associations within the normal population, our results may nonetheless be of clinical value in the context of eating disorders. The distributions of our main variables of interest suggest that those concepts are relatively endorsed in healthy individuals. By taking a continuous approach to clinical psychopathology, we argue that clinical populations differ from healthy populations by the degree of endorsement of those main variables of interest. To further explore whether high disgust propensity indeed sets people at risk via heightening self-disgust, a critical next step would be to investigate whether clinical participants as a group show both increased disgust propensity and heightened self-disgust.

Future studies are needed that further explore the involvement of different forms of disgust in body image. For example, it would be useful to investigate how pathogen disgust is involved in body image and in what way it is independent of self-disgust and other disgust-domains. In addition, some further clarification of the relationship between disgust propensity, body-related self-disgust and negative body image might be accomplished by examining those relationships across different demographic characteristics (e.g., age, gender). Importantly, it should be acknowledged that our studies relied on cross-sectional data; thus, on the basis of the current findings it can, for example, not be ruled out that in fact negative body image results in heightened self-disgust instead of vice versa. For firm conclusions about the direction of the relationships it would be necessary to use a longitudinal approach. Moreover, our design was correlational in nature; future studies using an experimental approach are needed to investigate the causal properties of the assumed relationships. In order to experimentally reduce self-disgust and therefore the assumed impact of self-disgust on body image, a paradigm in which disgust induced avoidance is prevented or opposed could be applied. In that way, one can also gain insight about the assumed relevance of disgust-induced avoidance in contributing to the perpetuation of self-disgust and negative body image.

### Conclusion

In sum, we did find consistent evidence for the involvement of self-disgust in the phenomenon of negative body image. More specifically, self-disgust was able to partly account for the finding that individuals with high trait disgust were more likely to experience a negative body image. Post-hoc speculations suggest that other forms of disgust-associations (in particular disgust towards disease-eliciting stimuli) might be involved in negative body image, relatively independently of self-disgust. Future research is needed to examine whether these relationships also extend to clinical levels of body image disturbance, and to clarify the causal role of different forms of disgust in the phenomenon of negative body image.

## Supporting information

S1 Appendix Regression residuals for all mediation analyses. (PDF)
