# Supplementary material for: Negative body image: Relationships with heightened disgust propensity, disgust sensitivity, and self-directed disgust
Source: PLoS One. 2018 Jun 5;13(6):e0198532. doi: 10.1371/journal.pone.0198532 (PMC5988313; doi:10.1371/journal.pone.0198532)

Regression Residuals for all Mediation Analyses

Study 1
Simple Mediation

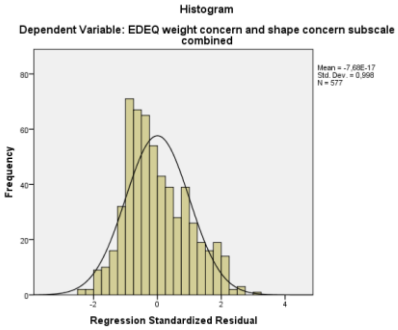

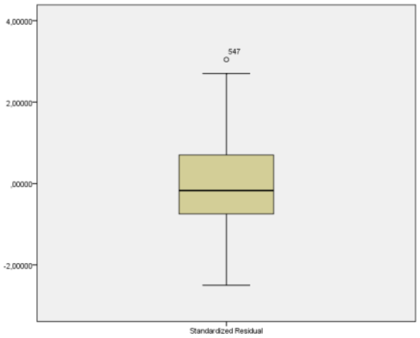

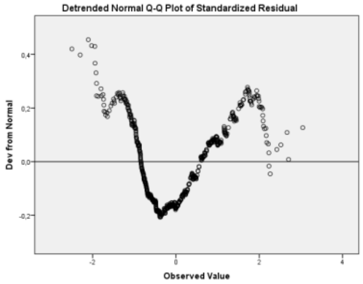

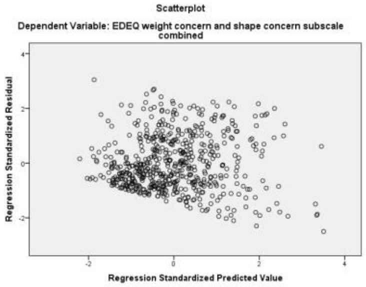

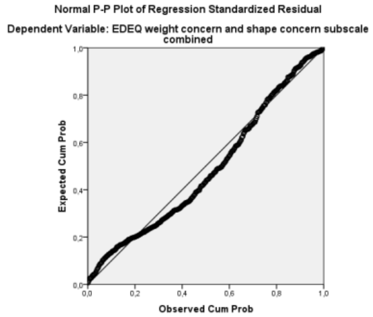

Study 1
Moderated Mediation

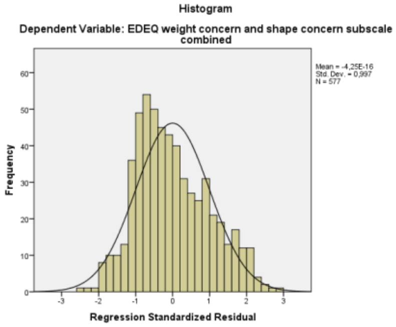

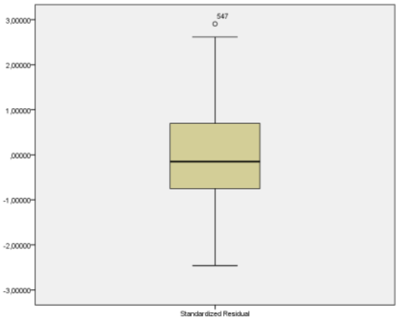

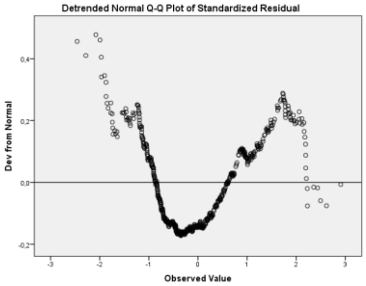

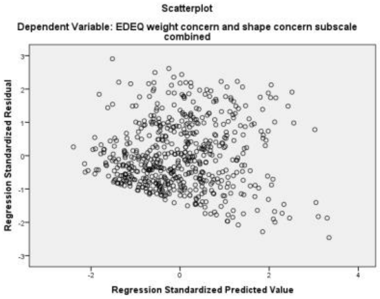

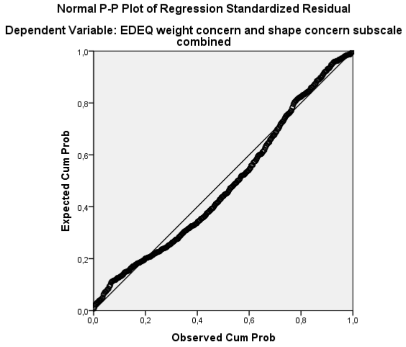

Study 2
Simple Mediation

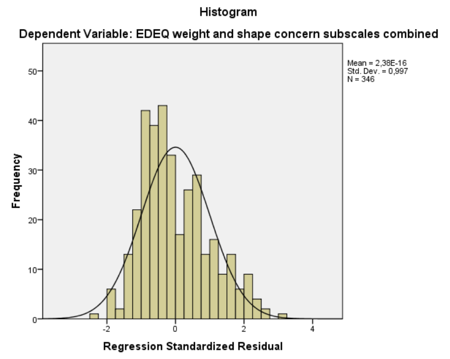

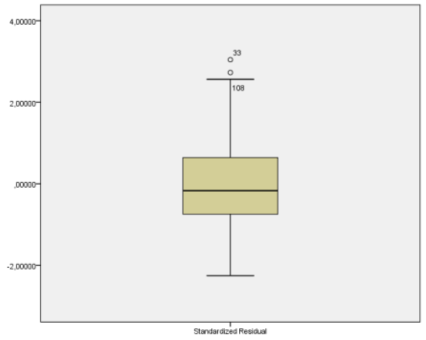

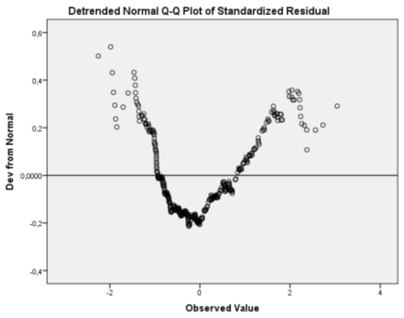

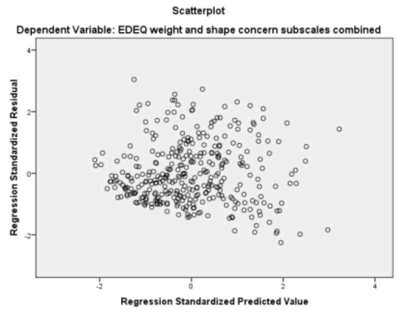

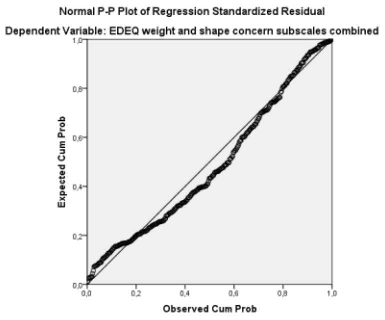

Study 2
Moderated Mediation

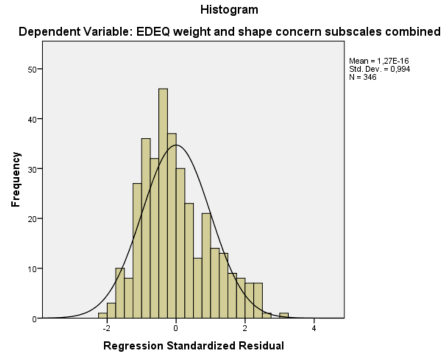

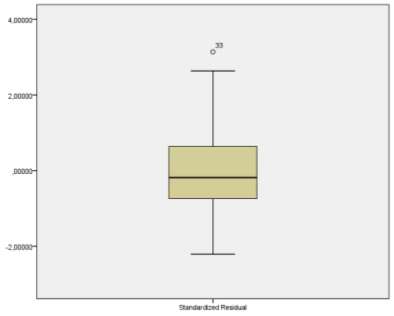

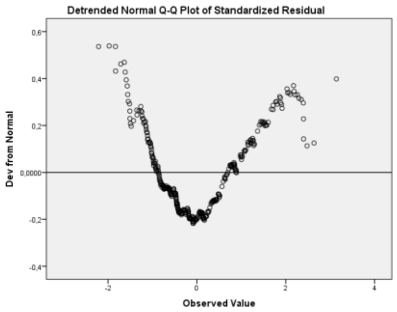

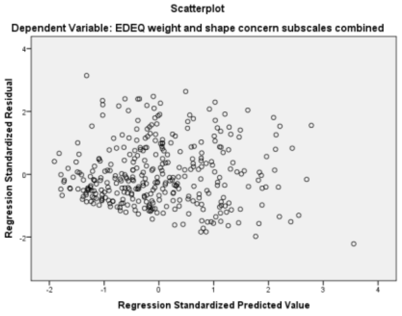

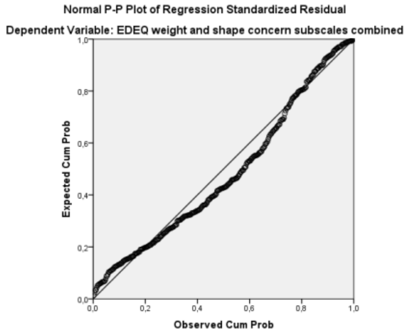

Supplement: S1 Appendix — (PDF) [file pone.0198532.s001.pdf]
